# UAVs Task and Motion Planning in the Presence of Obstacles and Prioritized Targets

**DOI:** 10.3390/s151129734

**Published:** 2015-11-24

**Authors:** Yoav Gottlieb, Tal Shima

**Affiliations:** Technion—Israel Institute of Technology, Technion City, Haifa 3200003, Israel; E-Mail: syoavgo@gmail.com

**Keywords:** UAV, task assignment, motion planning, obstacles, prioritized targets, Dubins car

## Abstract

The intertwined task assignment and motion planning problem of assigning a team of fixed-winged unmanned aerial vehicles to a set of prioritized targets in an environment with obstacles is addressed. It is assumed that the targets’ locations and initial priorities are determined using a network of unattended ground sensors used to detect potential threats at restricted zones. The targets are characterized by a time-varying level of importance, and timing constraints must be fulfilled before a vehicle is allowed to visit a specific target. It is assumed that the vehicles are carrying body-fixed sensors and, thus, are required to approach a designated target while flying straight and level. The fixed-winged aerial vehicles are modeled as Dubins vehicles, *i.e.*, having a constant speed and a minimum turning radius constraint. The investigated integrated problem of task assignment and motion planning is posed in the form of a decision tree, and two search algorithms are proposed: an exhaustive algorithm that improves over run time and provides the minimum cost solution, encoded in the tree, and a greedy algorithm that provides a quick feasible solution. To satisfy the target’s visitation timing constraint, a path elongation motion planning algorithm amidst obstacles is provided. Using simulations, the performance of the algorithms is compared, evaluated and exemplified.

## 1. Introduction

Unmanned vehicles are currently used in a variety of civil and military missions and are gradually replacing manned vehicles. The need for autonomous capabilities is derived from the fact that the number of unmanned vehicles used in each mission has increased dramatically, and the required collaboration between them for the successful completion of the mission cannot be achieved if each vehicle is operated individually. Furthermore, the complexity of the missions and the number of simultaneous actions to be performed may cause operator overload, which will lead to deterioration in the overall mission performance. In order to maximize performance, unmanned vehicles are expected to work together in coordination as a team. The overall team performance is expected to exceed the sum of the performances of the individual unmanned vehicles.

Two main aspects of the coordination and cooperation of a team of unmanned vehicles are path planning and assignment allocation, usually referred to as motion planning and task assignment problems, respectively. In the task assignment problem, a group of agents needs to be assigned to perform a number of tasks. The tasks can be performed by any of the group’s agents while minimizing or maximizing an objective function, depending on the scenario. An assignment task might be presented as a problem in graph theory [1], where the data in the graph are represented by vertices and edges. Such problems are commonly solvable using search algorithms by exploring the data structure level by level (breadth-first search) or reaching the leaf node first and backtracking (depth-first search).

The motion planning problem consists of planning a path for the vehicle while taking into account its kinematic and dynamic constraints, as well as generating feasible paths. The constraints may include a minimum turn radius and/or velocity limits, but there could also be obstacles scattered in the vehicle’s environment that need to be taken into consideration. In many cases ([2,3,4,5]), for the motion planning, the vehicle is modeled as a Dubins vehicle [6]: a vehicle moving in a plane while having a turn rate constraint. Extending the Dubins model with altitude control, time optimal paths between initial and final configurations are provided in [7]. Considering obstacles, motion planning algorithms for the Dubins vehicle are provided in [8,9,10,11,12]. In [13], a collision-free 3D motion planning algorithm is provided for an aerial vehicle. When using the Dubins model, the resulting trajectory is composed of straight lines and arcs of a minimum turn radius. Discontinuities in the curvature of the trajectory arise at the junctions between the line and arc segments, causing tracking errors when followed by an actual vehicle. To overcome such problems, an algorithm was proposed in works, such as [14,15], for generating a continuous-curvature path between an ordered sequence of waypoints (the junctions between the line and arc segments) produced by the motion planner.

The task assignment problem is usually coupled with that of motion planning, as the assignments allocation process depends on the path length, and the path length depends on the vehicle’s assignments. This coupling issue is addressed in the unmanned vehicles cooperative multiple task assignment problem (CMTAP) [16]. The CMTAP includes a scenario in which multiple unmanned vehicles perform multiple tasks on stationary targets. Different approaches based on customized combinatorial optimization methods were employed to solve this problem, including the mixed integer linear programming (MILP) [17,18], the capacitated transhipment network solver [19,20], genetic algorithms [16,21] and tree search methods [22,23]. In [19,21,24], timing and precedence constraints are also considered. In such scenarios, a target can be visited by a vehicle only if a specific task had first been performed on the target and a timing constraint was fulfilled. A method to elongate minimum distance paths for constant speed vehicles to meet the target timing constraints is presented in [25]. The presented works account for the vehicles’ constraints, but they simplify the problem by assuming that the environment is obstacle free. Most of the studies that take into account obstacles address only the motion planning subproblem between the initial and final configuration. They include methods such as the rapidly-exploring random trees (RRT) method [26], probabilistic roadmaps [27] and the kinodynamic method [28].

One of the main properties of the problem stated above is the assumption that the targets have the same characteristics and differ only in their position. In many scenarios, each target has unique attributes, which include different importance and priority. The targets’ priority may also vary in time depending on the specific scenario. Cases in which targets are assigned with a priority value were studied in [29,30,31]. The targets’ priority was addressed by using an objective function, which includes a constant parameter describing the priority value. In these works, the vehicles’ constraints were not taken into account, and the environment was assumed to be free of obstacles, which may lead to infeasible trajectories.

In this paper, the task assignment problem coupled with the problem of motion planning for a team of fixed-winged unmanned aerial vehicles that needs to service (fly over) multiple targets, while taking into account the vehicles’ kinematic constraints and the need to avoid obstacles scattered in the environment, is addressed. It is also assumed that vehicles carry downward pointing body-fixed sensors and, thus, are required to approach a target flying straight and level. The main contribution of this paper is incorporating these constraints together with the targets’ priority to create a more realistic time-varying priority scenario and by proposing a path elongation algorithm, used to consider the targets’ timing constraints dictated by the different scenarios’ characteristics. In order to solve this coupled problem, it is represented as a decision tree, and two tree search algorithms are proposed.

The remainder of this paper is organized as follows: In Section 2, a mathematical formulation of the problem is given. Section 3 describes the motion planning subroutine used. In Section 4, a solution to the task assignment problem is proposed. In Section 5, the simulation results of different sample runs are provided, and concluding remarks are offered in Section 6.

## 2. Problem Formulation

The problem considered in this work includes allocating a group of fixed-winged aerial vehicles to a given set of targets, while taking into account the vehicles’ kinematic constraints and avoiding collision with obstacles scattered in the environment. It is assumed that vehicles carry downward pointing body-fixed sensors and, thus, are required to approach a target flying straight and level. Each target is assigned with a time-dependent value (referred to as the target benefit) that represents the target’s importance and priority. The objective is to maximize a reward function, which is the sum of all of the benefits gathered by the group of vehicles.

### 2.1. Example Scenario

The motivation for solving this problem can be explained using the following example: A network of unattended ground sensors (UGS) and a team of unmanned vehicles are used to prevent intruders’ access to a restricted zone (base defense) [32]. The UGS network is deployed at critical road junctions, and when a sensor is triggered by an intruder, the location is sent as a target to be visited by the team of unmanned vehicles. If there are multiple intrusions at different times, the group of vehicles must be allocated according to the vehicles’ response time (time to target) and the target’s priority, which can be based on the order of the UGS triggering time or on the location of the sensor. The target (sensor) priority is time dependent, since, as time passes, the intruder may advance to a different location, and the target relevance decreases. Additionally, the unmanned vehicle may have a timing constraint for visiting the target, e.g., only after it has been classified and cleared from friendly forces by the ground forces. This introduces a timing constraint that needs to be considered when allocating targets to the team of vehicles.

### 2.2. Vehicles

**Figure 1:**
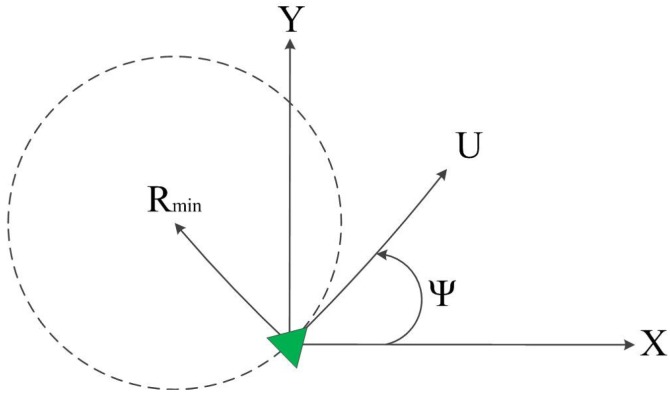
Vehicle kinematics.

Let $V=\{V_{1},V_{2},\ldots ,V_{N_{V}}\}$ be a set of unmanned aerial vehicles (UAVs) that need to complete the visit requirements of the given set of targets. The vehicles have a minimum turn radius and can move only forward at constant speed. The kinematic constraints need to be accounted for when planning the vehicles’ trajectory. The equations of motion are presented below:

Vehicle kinematics:
  (1) $$\begin{array}{ccc} \dot{x} & = & U\cos\psi \\ \dot{y} & = & U\sin\psi \\ \dot{\psi } & = & \omega \end{array}$$
Turn rate constraint (given a minimum turn radius):
  (2) $$\;|\omega |⩽U/R_{\min}$$

where (x,y) are the vehicle’s Cartesian coordinates, *ψ* is the vehicle’s orientation angle and *U* and *ω* are the vehicle’s constant speed and turn rate, respectively. A schematic planar view of the vehicle’s kinematics is presented in Figure 1. It should be noted that the above kinematics may also represent the motion of other types of vehicles moving in a plane, such as ground vehicles.

The set of initial conditions that represents the vehicles’ initial position and orientation is given by $V_{IC}=\left\{\left(x_{1_{0}},y_{1_{0}},\psi_{1_{0}}\right),\left(x_{2_{0}},y_{2_{0}},\psi_{2_{0}}\right),\ldots ,\left(x_{N_{V_{0}}},y_{N_{V_{0}}},\psi_{N_{V_{0}}}\right)\right\}$.

### 2.3. Body-Fixed Sensors

**Figure 2:**
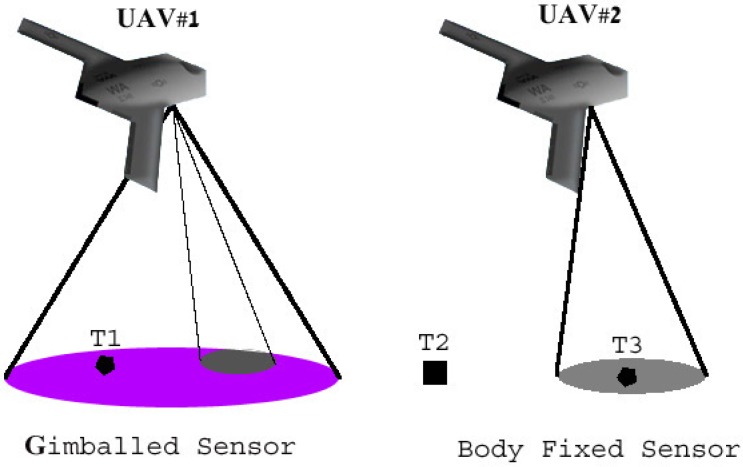
Sensor footprint schematic examples.

UAV sensors can be roughly divided into two categories: gimballed and body fixed. Gimballed sensors are usually more complex and enable pointing the sensor to a desired position, with usually minimal effect of the UAV’s state. Body-fixed sensors are usually much simpler and less expensive, but their footprint is determined by the UAV’s states, such as pitch and roll angles. Figure 2 [33], presents a schematic example of the footprints of gimballed and body-fixed sensors. In the figure, UAV#1 is carrying a gimballed sensor, which can be moved within a larger possible footprint. Target 1 (T1) is enclosed by this larger possible footprint, but the sensor is currently pointing to a different direction. UAV#2 is carrying a body-fixed sensor, and Target 3 (T3) is within its footprint; but, its tracking will not be assured if the UAV rolls or pitches. Target 2 (T2) is outside the footprints of both UAVs.

In this research, it is assumed that the fixed-winged UAVs carry body-fixed sensors that point directly downwards. Consequently, to ensure that the designated target will be inside the sensor’s field-of-view, it is required that the UAVs approach a target flying straight and level. This ensures that the UAVs do not bank before crossing the target.

### 2.4. Obstacles

Let Ω be the two-dimensional physical environment in which the vehicles move ((x,y)∈Ω) and the targets are located. Let O⊂Ω be the set of obstacles that the vehicles need to avoid. O is considered to be a set of disjoint convex polygons. It is required that:

(3) (x(t),y(t))∩int(O)=∅

In this work, it is assumed that a vehicle is allowed to graze the obstacles’ boundaries, but it cannot penetrate them. In reality, the obstacles considered by the algorithm would be slightly larger in size than the actual obstacles, so as to ensure that the vehicle is safe, even if the algorithm requires the vehicle to take a path that grazes an obstacle boundary.

### 2.5. Targets and Benefits

Let $T=\{T_{1},T_{2},\ldots ,T_{N_{T}}\}$ be the set of $N_{T}$ stationary targets, located in Ω, designated to the group of fixed-winged unmanned aerial vehicles. It is assumed that the minimum distance between each pair of targets is larger than $2R_{min}$.

The set of timing constraints assigned to each target is given by $tc=\{tc_{1},tc_{2},\ldots ,tc_{N_{T}}\}$. The vehicle is allowed to visit and perform its given task at the target only if the time required for a vehicle to arrive from its initial configuration $\left(x_{0},y_{0},\psi_{0}\right)$ to the target is greater than or equal to the time constraint specified for the relevant target.

Let $C=\{C_{1},C_{2},\ldots ,C_{N_{T}}\}$ be the set of initial benefits assigned to each target, and let $S=\{1,2,\ldots ,N_{T}\}$ be the set of stages in which a target is allocated as an assignment to a vehicle. The set of stages *S* is used to keep track of the vehicles’ assignments history, which is of high importance when calculating the vehicles’ path length. Furthermore, each stage corresponds to a layer in the tree representation used in Section 3.1.

The target’s benefit represents the value granted to a vehicle for visiting the target. Since the benefits are time dependent, a mathematical formulation, which is referred to as the “benefit function”, is proposed. This formulation represents the reward granted to a vehicle for visiting a target, depending on the target’s priority (represented by its initial benefits) and the time required for a vehicle to arrive at the target from its initial position.

Let $x_{ik}^{m}\in \left\{0,1\right\}$ be a binary decision variable that equals one if vehicle i∈V visits target k∈T at stage m∈S and is zero otherwise, and let $X_{m}=\left\{x_{ik}^{1},x_{ik}^{2},\ldots ,x_{ik}^{m}\right\}$ be the set of assignments up to and including stage *m*.

Let:

(4) $$t_{ik}^{m}=L_{ikm}^{X_{m-1}}/U$$

be the time required for vehicle i∈V to travel to target k∈T at stage m∈S. This time is obtained through the division of the vehicle *i* path length to target *k* at stage *m*, notated as $L_{ikm}^{X_{m-1}}$, by its constant speed. Note that $L_{ikm}^{X_{m-1}}$ depends on the position and orientation of vehicle *i* before stage *m*, which, in turn, depends on the initial position and orientation of the vehicle and the targets it visited in the subsequent stages until stage *m*. The assignment history prior to stage *m* is included in the vehicle path length expression by the notation $X_{m-1}$.

The benefit function, which represents the reward granted to vehicle *i* for visiting target *j*, is formulated as follows:

(5) $$\;C_{j}e^{-A\sum_{m=1}^{l}\sum_{k=1}^{N_{T}}t_{ik}^{m}x_{ik}^{m}}$$

where *A* is a user-defined coefficient, which defines the benefit function’s descent rate, and:

(6) $$\sum_{m=1}^{l}\sum_{k=1}^{N_{T}}t_{ik}^{m}x_{ik}^{m}$$

is the time required for vehicle *i* to arrive from its initial configuration $\left(x_{i_{0}},y_{i_{0}},\psi_{i_{0}}\right)$ to target *j* that is visited at stage *l*.

**Figure 3:**
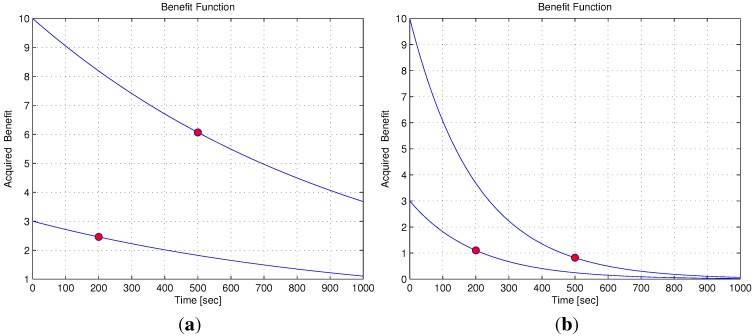
Benefit function. (**a**) Benefit function over time; (**b**) benefit function over time, increased decent rate.

This formulation helps create a problem in which the vehicle assignments’ order depends on the path to each target and not only on the target’s initial priority (for example, the highest priority target is not necessarily visited first, and the time to arrive at the target’s location is also taken into consideration). In addition, the same formulation can be used to describe the example described in Section 2.1. The example includes a UGS network and a team of unmanned vehicles used for intruder detection and identification. The vehicles’ response time is taken into account by calculating the vehicles’ path length, and the targets’ different initial priority represents the order of the UGS triggering time. Since the time it takes a vehicle to reach a target depends on the vehicle’s path length, the latter will be calculated using a motion planning subroutine, described in Section 3. Figure 3a shows the benefit function change over time, each curve beginning with a different initial value (initial benefits three and 10). The benefit function is a monotonically-decreasing function, and as such, the initial value diminishes as time progresses. When the descent rate coefficient is changed (increased by five times), the benefit rapidly diminishes over time, as seen in Figure 3b. The increase of the descent rate may also cause a change in the targets assigned to each vehicle or a different order in which the assigned targets need to be visited.

### 2.6. Cost Function

The objective is to complete the visit requirement (visiting the given set of targets once) so as to maximize a reward function. The reward function considered is the overall benefits acquired by the vehicles:

(7) $$\;J_{1}=\sum_{i=1}^{N_{V}}\sum_{l=1}^{N_{T}}\sum_{j=1}^{N_{T}}\left[C_{j}e^{-A\sum_{m=1}^{l}\sum_{k=1}^{N_{T}}t_{ik}^{m}x_{ik}^{m}}\right]x_{ij}^{l}$$

where $x_{ij}^{l}\in \left\{0,1\right\}$ is a binary decision variable that equals one if vehicle *i* visits target *j* at stage *l*. Since the benefit function diminishes with time, a “lost” benefit function is formulated and is given by $C_{j}-C_{j}e^{-A\sum_{m=1}^{l}\sum_{k=1}^{N_{T}}t_{ik}^{m}x_{ik}^{m}}$. The “lost” benefit function formulation allows the definition of a cost function, which is the equivalent to the reward function defined above, but instead of maximizing the reward function, the objective is to minimize the cost function. The cost function mathematical formulation is given by:

(8) $$\;J_{2}=\sum_{i=1}^{N_{V}}\sum_{l=1}^{N_{T}}\sum_{j=1}^{N_{T}}\left[C_{j}-C_{j}e^{-A\sum_{m=1}^{l}\sum_{k=1}^{N_{T}}t_{ik}^{m}x_{ik}^{m}}\right]x_{ij}^{l}$$

The constraints of the problem are given by:

(9) $$\begin{array}{ccc} & & \sum_{l=1}^{N_{T}}\sum_{i=1}^{N_{V}}x_{ij}^{l}=1,\;j=1,\ldots ,N_{T} \end{array}$$

(10) $$\begin{array}{ccc} & & \sum_{i=1}^{N_{V}}\sum_{j=1}^{N_{T}}x_{ij}^{l}=1,\;\forall \;l=1,\ldots ,N_{T} \end{array}$$

(11) $$\begin{array}{ccc} & & \sum_{m=1}^{l}\sum_{k=1}^{N_{T}}t_{ik}^{m}x_{ik}^{m}\geq tc_{j},\;\forall \;j=1,\ldots ,N_{T},\;tc_{j}\in tc,\;s.t.\;x_{ij}^{l}=1 \end{array}$$

Equation (9) ensures that each target is visited once. Equation (10) ensures that only a single vehicle is assigned to a target in each stage. In Equation (11), the timing constraint is posed. The time required for a vehicle to arrive at the target location from its initial configuration must be greater than or equal to the time constraint dictated as part of the problem’s initial parameters. If the vehicle’s arrival time at the target is less than the time constraint $tc_{j}$, the path should be elongated, otherwise the third constraint will be violated.

In [16,21,23], somewhat similar problems involving multiple targets and vehicles were solved. The cost function used in the related works is the sum of the path lengths of all of the vehicles and can be formulated as:

(12) $$\;J_{3}=\sum_{i=1}^{N_{V}}\sum_{l=1}^{N_{T}}\sum_{j=1}^{N_{T}}L_{ij}^{l}x_{ij}^{l}$$

In these cases, the targets’ importance is identical and ignored when solving the problem. In the simulation results’ section, this cost function is used to help compare the performance of the proposed algorithms.

The solution process of the problem includes solving two integrated subproblems: task assignment and motion planning problems. To minimize the cost function, the task assignment depends on the underlying motion planning for the path length, while the motion planning depends on the task assignment for the order of the vehicle’s targets. This makes the problems coupled.

## 3. Motion Planning

For the motion planning problem, it is assumed that each vehicle is assigned a list of an ordered set of targets, made by the task assignment algorithm. The goal of the motion planning is to derive a trajectory for each vehicle to visit all of the targets on the list, avoid collision with obstacles and respect the vehicle kinematic constraints (described in Section 2.2) and the timing constraint (described in Equation (11)). Due to the sensor-oriented requirement of having the vehicles fly straight and level when approaching a target (see Section 2.3), the planner needs to issue a trajectory with straight line segments preceding the arrival of a vehicle to a target.

The motion planning problem description and solution presented below is based on the study described in [12]. Since the targets’ timing constraints are not included in [12], a path elongation algorithm is proposed in Subsection 3.2.

### 3.1. Tree Formulation

In order to represent the motion planning problem in the form of a decision tree, it is necessary to generate nodes representing the following: targets position, vehicle’s initial configuration and obstacles’ vertices (under the assumption of polygonal obstacles). The vehicle’s path will either be a direct path (free of obstacles) connecting the initial configuration and the set of targets, or a path that also passes through some of the obstacles’ vertices, in case a direct path does not exist. Each branch of the tree represents the described path. The root node (initial configuration) is connected to all of the target nodes, and if a direct path is not feasible, obstacles nodes are also included. The goal is to find the branch that provides the minimum time path. In order to to satisfy the timing constraint described in Equation (11), a path elongation is provided in the following section.

The vehicles in this work are modeled as Dubins vehicles. The Dubins path is a concatenation of arcs of minimum radius turn and straight line segments that connect an initial and final configuration (position and orientation). The optimal path can be achieved by checking six possible path types for the Dubins vehicle [6]. If the orientation angle in the final configuration is removed, the number of possibilities is reduced. This is known as the relaxed Dubins path that include only four possibilities [34].

**Figure 4:**
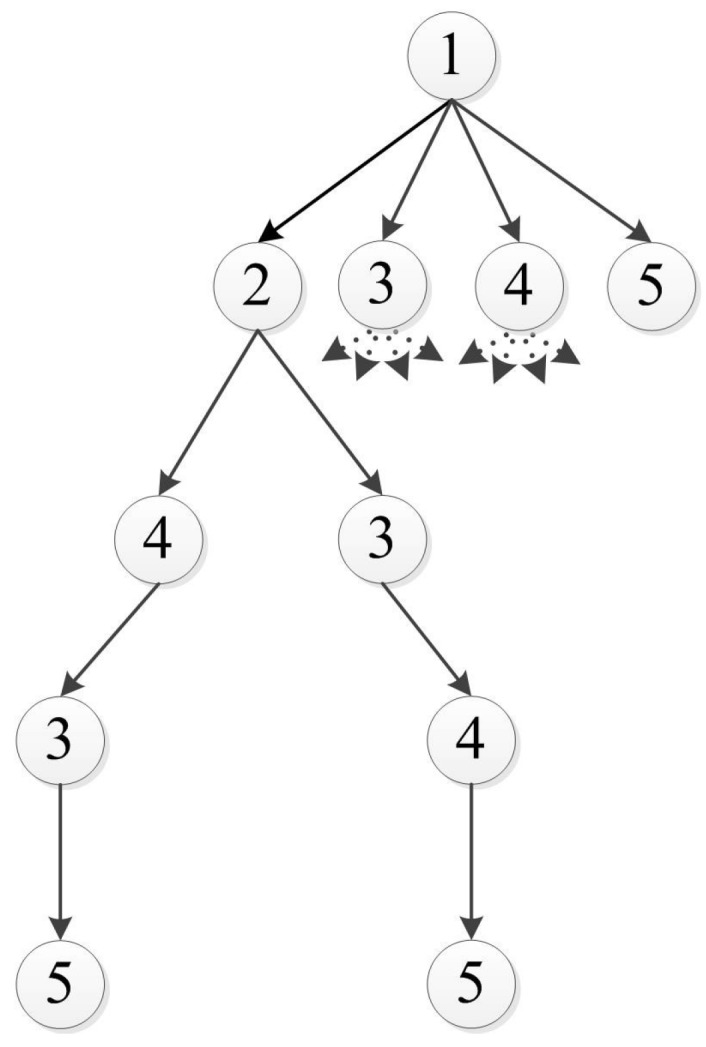
An example of a five-configuration tree.

An important benefit obtained by using the relaxed path is explained using the example given in Figure 4. When calculating the optimal path between an initial (Node 1), final (Node 5) configurations and three additional unordered configurations (for example: obstacle vertices) located between them (Nodes 2–4), the following branches of the tree graph are generated: A branch connecting nodes 1-2-3-4-5 and a branch connecting nodes 1-2-4-3-5. In the relaxed case, the path connecting Nodes 1 and 2 should be calculated only once, as it is independent of the remaining nodes. However, in the non-relaxed case, the arrival angle at Node 2 depends on the order of the following nodes (Node 3 or 4), and the path between Nodes 1 and 2 needs to be calculated separately for each branch. This attribute, where the path between two nodes does not depend on the following nodes, enables us to pose the problem as a tree.

Moreover, as the minimal distance between each of two targets is larger than two times the minimum turn radius of the vehicles, it is guaranteed that the optimal relaxed paths between each pair of targets consist of a terminal straight line segment, satisfying the body-fixed sensor-originated requirement that a UAV approaches a target flying straight and level, discussed in Section 2.3.

In order to find the relaxed optimal path that connects the initial configuration and the targets’ set and does not intersect with obstacles, it is necessary to search the tree presented above. The search process includes calculating the relaxed path connecting the different graph nodes: obstacles’ vertices or targets’ positions. In this search process, the calculation of the relaxed path is repeated multiple times, especially in large-scale scenarios. When real-time scenarios are considered, the use of the relaxed path becomes highly beneficial, as the computational complexity is significantly reduced, compared to the non-relaxed case.

In this work, an existing motion planning algorithm is used to find an admissible path of bounded curvature through a given ordered set of points among obstacles without timing constraints. The motion planning solution can be achieved by one of two algorithms: (1) an exhaustive algorithm, which explores every branch of the search tree and evaluates every possible visit order sequence in order to find the minimum cost one (See Algorithm B1 in the Appendix); and (2) an A* like heuristic algorithm, which uses Euclidean distances as a heuristic estimation and a greedy approach to find a feasible path (See Algorithm A1 in the Appendix). Since the existing algorithms do not take into account the timing constraints when calculating the vehicles’ trajectory, a path elongation algorithm amidst obstacles is proposed in the following section. The combined motion planning algorithm and the path elongation algorithm are used as a subroutine for the developed task assignment algorithm.

### 3.2. Path Elongation Algorithm

A path elongation algorithm, used to lengthen a vehicle’s path that connects an initial configuration and a target position in an environment with obstacles, is now presented. Different methods can be used to elongate the vehicle path. For example, a different (higher cost) branch of the tree can be used instead of the current branch. Another possibility is to increase the vehicle’s turn radius to elongate the path. These methods, however, proved to be inefficient in terms of computational run time.

The path elongation method used in this paper is based on appending loitering circles. The technique forces the vehicle to perform a circular flight with a minimum turn radius around a specific point until the timing constraint is fulfilled. One of the motion planning algorithms is used to generate the vehicle’s path. If the timing constraint is not fulfilled, the path elongation algorithm is employed.

The algorithm is given in Algorithm 1. The inputs to the algorithm are the vehicle’s configuration and speed, obstacle vertices’ locations, target position, time constraint and the vehicle’s current path, the order and orientation in which nodes are visited. The output is the vehicle’s trajectory in terms of the order in which nodes are to be visited, the node in which the loitering circles are performed and the number of cycles.

**Algorithm 1** Path elongation algorithm amidst obstacles.
**Input:** Initial position and orientation of vehicle; vehicle speed; obstacle information; target position; path nodes position and orientation; path length; time constraint;
**Output:** Vehicle trajectory (visit order and elongation circles node if needed)1:EvList ← generate a list of all elongation vertices in the environment2:DpList ← Construct a sorted list of direct Dubins path vertices list3:**if** EvList ∩ pathNodes ≠ ∅
**then**4:     elongationVertex ← EvList ∩ pathNodes5:     elongationNode ← elongationVertex(1)6:     loiterCirclesNumber ←⌈(timeConstraint-(pathLength/VehicleSpeed))/2*π*$R_{min}$⌉7:     newVisitOrder ← path nodes8:     **return** newVisitOrder, elongationNode, loiterCirclesNumber9:**end**
**if**10:**for** iPathNodes = 1 to nPathNodes **do**11:     intersectionCounter← checkCirclesIntersection[iPathNodes, obstaclesInfo]12:     **if** intersectionCounter ≠ ∅
**then**13:         elongationNode ← pathNodes(iPathNodes)14:         loiterCirclesNumber ←⌈(timeConstraint-(pathLength/VehicleSpeed))/2*π*$R_{min}$⌉15:         newVisitOrder ← path nodes16:         **return** newVisitOrder, elongationNode, loiterCirclesNumber17:     **end**
**if**18:**end**
**for**19:**if** DpList ∩ EvList ≠ ∅
**then**20:     newDplist=DpList ∩ EvList21:     **for** i = 1 to newDplistLength **do**22:         [newVisitOrder, newPathLength, newPathNodesAngles, intersectionCounter] ←
         motionPlanningAlgo[vehicleConfiguration, newDplist(i), target, obstaclesInfo]23:         **if** intersectionCounter=∅
**then**24:            elongationNode ← newDplist(i)25:            **if** timeConstraint > newPathLength/VehicleSpeed **then**26:                loiterCirclesNumber ←⌈(timeConstraint-(newPathLength/VehicleSpeed))/2*π*$R_{min}$⌉27:            **end**
**if**28:            **return** newVisitOrder, elongationNode, loiterCirclesNumber29:         **end**
**if**30:     **end**
**for**31:**end**
**if**32:sortedEvList ← euclideanSort[EvList]33:**for** i = 1 to sortedEvListLength **do**34:     [newVisitOrder, newPathLength, newPathNodesAngles, intersectionCounter] ←
     motionPlanningAlgo[vehicleConfiguration, sortedEvList(i), target, obstaclesInfo]35:     **if** intersectionCounter=∅
**then**36:         elongationNode ← sortedEvList(i)37:         **if** timeConstraint > newPathLength **then**38:            loiterCirclesNumber ←⌈(timeConstraint-(newPathLength/VehicleSpeed))/2*π*$R_{min}$⌉39:         **end**
**if**40:         **return** newVisitOrder, elongationNode, loiterCirclesNumber41:     **end**
**if**42:**end**
**for**

Since there are obstacles scattered in the environment, the vehicle cannot perform loitering circles around any given point without intersecting with the obstacles. Let us define a set of points that will be referred to as “elongation vertices”. This set includes obstacles’ vertices in which the vehicle can perform a loiter circle (of minimum turn radius) without intersecting with the obstacles boundaries for any given feasible orientation; infeasible orientations are defined as angles in which the vehicle cannot be positioned without penetrating the obstacle boundary and, therefore, are initially not included in the set of arrival angles, forming the vehicle’s path.

It should be noted that, as discussed in Section 2.4, a vehicle is allowed to graze the obstacles’ boundaries, but it cannot penetrate them. In reality, the obstacles considered by the algorithm would be slightly larger in size than the actual obstacles, so as to ensure that the vehicle is safe, even if the algorithm requires the vehicle to take a path that grazes an obstacle boundary. Thus, it is safe for the vehicles to perform loitering circles that graze an obstacle edge.

**Figure 5:**
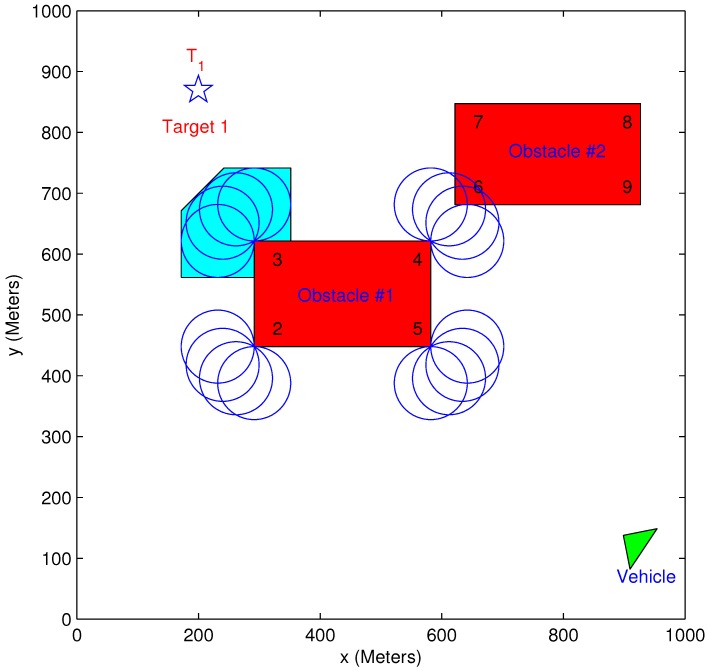
Elongation vertices example: Minimum turn radius = 60 m.

**Figure 6:**
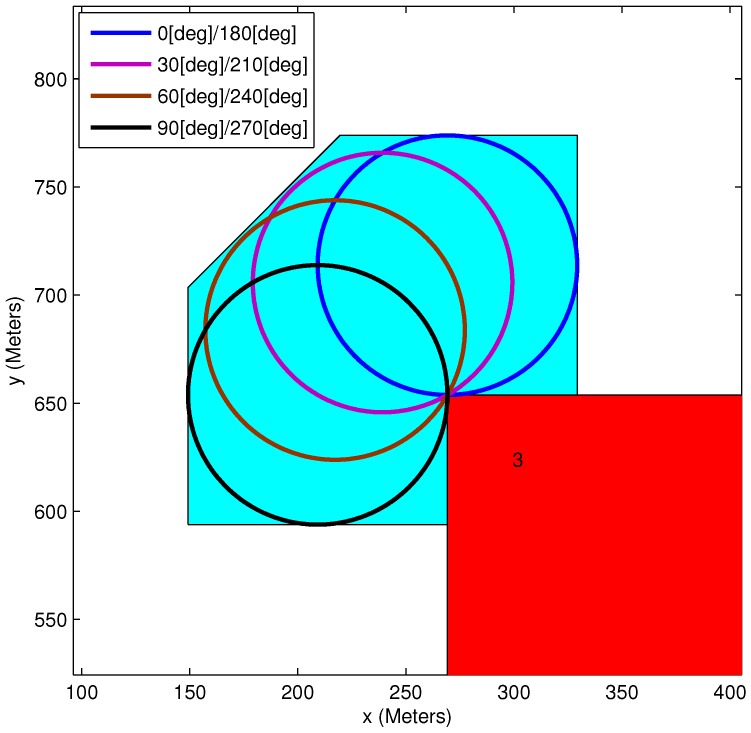
Elongation vertex: $30^{\circ }$ discretization.

An example of the elongation vertices is given in Figure 5. This example includes a vehicle, a target and two rectangular obstacles. On each of Obstacle 1’s vertices, loitering circles are drawn. Each circle corresponds to a specific feasible vehicle orientation (a $30^{\circ }$ discretization of the feasible angles’ range was used in this example). As can be seen, Vertex 3 can be defined as an elongation vertex, while Vertex 4 is not part of the this set, as several loitering circles in this vertex intersect with Obstacle 2. A zoom-in of Vertex 3 is presented in Figure 6. The discretization of the orientation angles is clearly visible, with each circle corresponding to two feasible angles. All of the loitering circles in each vertex can be enclosed by a polygon, as presented in the figures.

**Figure 7:**
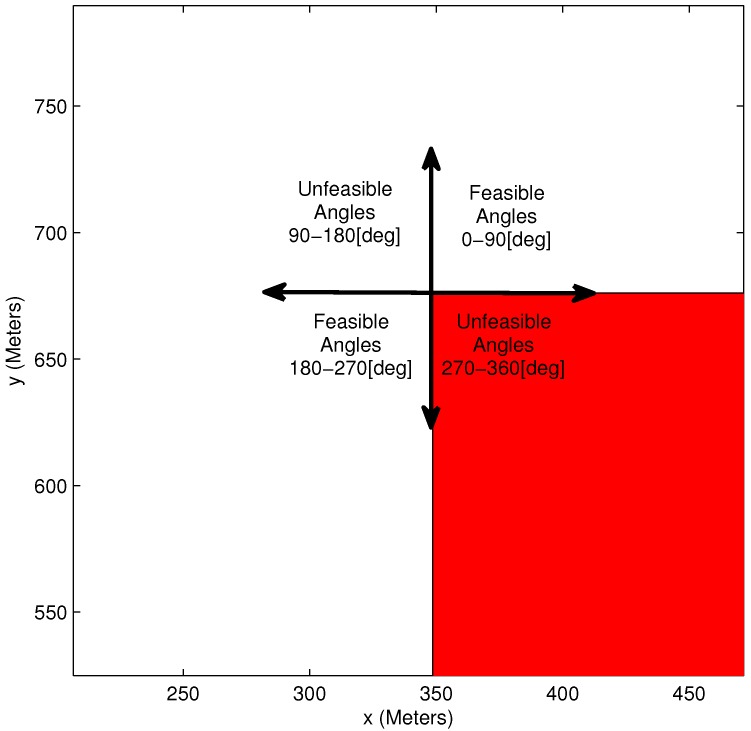
Feasible and unfeasible angles example.

Figure 7 shows the range of infeasible/feasible orientation angles at a specific obstacle vertex. It is important to notice that each of the rectangle obstacle’s vertices has a different distribution of infeasible/feasible orientation.

The key idea behind the proposed algorithm is to locate a node of the input path that is also an elongation vertex or to generate a new path that includes at least one elongation vertex. If a new path is generated and the timing constraints are not satisfied, the vehicle can perform the required amount of loitering cycles at the elongation vertex.

## 4. Task Assignment

**Figure 8:**
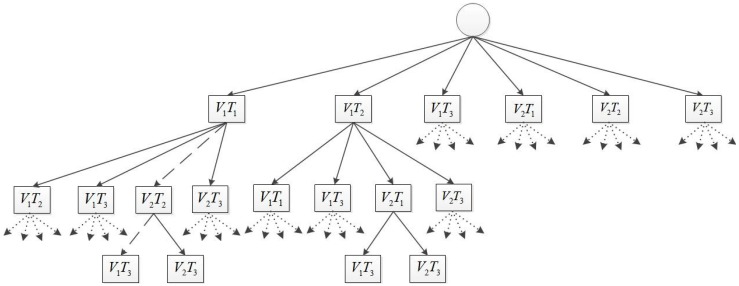
A tree for two vehicles and three targets.

The task assignment solutions are now provided. First, the problem is presented as a tree (as can be seen in Figure 8) by generating nodes that describe a vehicle $V_{i}$ assigned to a target $T_{j}$. Figure 8 presents a search tree for a scenario in which three targets need to be assigned to two vehicles. For a concise illustration, only some of the branches of the tree are shown. The branch shown by a dashed line gives an assignment $V_{1}T_{1},V_{1}T_{3},V_{2}T_{2}$. This means that target $T_{2}$ is assigned to vehicle $V_{2}$, and targets $T_{1}$ and $T_{3}$ are assigned to vehicle $V_{1}$, which must visit the assigned targets in that specific order. Each node of the tree is associated with a cost. For example: node $V_{i}T_{j}$ has a cost that equals the “lost” benefit granted to vehicle *i* for visiting target *j*. The “lost” benefit value depends on the time that it takes vehicle *i* to reach target *j* from its initial position, which depends on the vehicles’ path length. The path is obtained using a motion planning subroutine (described in Section 3), which guarantees a feasible path for the vehicles. Since the motion planning subroutine is used in the task assignment process, the problem solution consists of a primary task assignment tree search, which depends on a secondary motion planning tree search.

Two algorithms that provide solutions to the task assignment problem are proposed, an exhaustive search algorithm and a greedy algorithm. The greedy algorithm provides a computationally-fast solution that may not be optimal, and the exhaustive algorithm explores all of the assignment possibilities to derive an assignment’s allocation with the minimum cost value. The main use of the greedy algorithm may be in scenarios where the assignment cannot be planned beforehand and needs to be planned in real time. Such cases arise when new targets pop-up during the engagement and/or other relevant changes occur in the scenario (like a loss of a vehicle).

### 4.1. Exhaustive Task Assignment Algorithm

The proposed algorithm that is described in Algorithm 2 explores every branch of the tree to evaluate all of the assignment’s possibilities.

The inputs to the algorithm are the initial configuration and constant speed of the vehicles, the locations of the target points, information about the obstacles and the time constraint of each target.

The algorithm’s first step includes generating an upper bound on the cost (Line 1), calculated by using the greedy algorithm described in Section 4.2. This upper bound will be useful to bound the branching of the tree, thus preventing unnecessary explorations. Then the following lists are initialized (Lines 2–5). A TargetsList variable is constructed that includes all of the targets that need to be visited by the vehicles, and the corresponding time constraints are entered into a TimeConstraint list. Furthermore, a vehicleTargetList that contains the assigned targets (and order of visit) of each vehicle and an OpenSet that stores all of the nodes to be examined are initialized to an empty set.

Next, the first layer of the tree (as presented in Figure 8) is generated. All possible vehicle-target pair combinations are described as nodes and added to OpenSet for further exploration (Lines 6–26). Each node’s vehiclePath and vehicleTargetsList fields are initialized to zero and empty vectors, respectively (Line 8). Then, the current target ($T_{j}$) is assigned to the current vehicle ($V_{i}$) (Lines 9–10), and the value of the path length and the cost are updated in the corresponding position (*i*-th entry) (Line 11, Line 15).

**Algorithm 2** Task assignment exhaustive search algorithm.
**Input:** Vehicles’ initial configuration (*V*) and constant speed, targets’ position and their visit requirements (*T*), obstacle vertices’ positions, targets’ time constraints (tc)
**Output:** Assignment for each vehicle and the order in which the assigned targets needs to be visited1:UpperBound ← greedy task assignment algorithm solution2:TargetsList ← $\{T_{j},\;j=1,\ldots ,N_{T}$}3:TimeConstraint ← $\{tc_{j},\;j=1,\ldots ,N_{T}$}4:vehicleTargetList(*i*) ← [ ], $i=1,\ldots ,N_{V}$5:OpenSet ← [ ]6:**for**
$V_{i},\;i=1,\ldots ,N_{V}$
**do**7:     **for**
$T_{j}\in$ targetsToVisit **do**8:         node.vehiclePath(*k*) ← 0 , node.vehicleTargetsList(k) ← [ ], $k=1,\ldots ,N_{V}$9:         node.vehicle ← $V_{i}$10:         node.vehicleTargetsList(i) ← $T_{j}$11:         node.vehiclePath(i)=PathLength$\left(V_{i},T_{j}\right)$12:         **if** TimeConstraint$\left(T_{j}\right)$ < (node.vehiclePath(i)/VehicleSpeed$\left(V_{i}\right)$) **then**13:            node.vehiclePath(i)=PathElongation$\left(V_{i},T_{j}\right)$14:         **end**
**if**15:         node.cost ← lostBenefitFunction(PathLength$\left(V_{i},T_{j}\right)$)16:         node.targetsList ← targetsList$\setminus T_{j}$17:         **if** node.targetsList =∅
**then**18:            **if** Cost(node) ≤ UpperBound **then**19:                UpperBound ← Cost(node.cost)20:                vehicleTargetsList(*i*) ← node.vehicleTargetsList(i)21:            **end**
**if**22:         **else**23:            OpenSet ← OpenSet ∪ node24:         **end**
**if**25:     **end**
**for**26:**end**
**for**27:**while** OpenSet ≠∅
**do**28:     parentNode ← OpenSet(last entered node) – depth-first search29:     **for**
$V_{i},\;i=$ parentNode.vehicle$,\ldots ,N_{V}$
**do**30:         **for**
$T_{j}\in$ targetsToVisit **do**31:            childNode.cost(*k*) ← parentNode.cost(*k*),
             childNode.vehicleTargetsList(k) ← parentNode.vehicleTargetsList(k),
             childNode.vehiclePath(k) ← parentNode.vehiclePath(k), $k=1,\ldots ,N_{V}$32:            **if** TimeConstraint$\left(T_{j}\right)$ < (childNode.vehiclePath(i)
                                             +PathLength$\left(V_{i},T_{j}\right)$)/VehicleSpeed$\left(V_{i}\right)$
**then**33:                PathLength$\left(V_{i},T_{j}\right)$=PathElongation$\left(V_{i},T_{j}\right)$34:            **end**
**if**35:            childNode.vehiclePath(i)=childNode.vehiclePath(i) + PathElongation$\left(V_{i},T_{j}\right)$36:            childNode.cost(*i*) ← lostBenefitFunction(childNode.vehiclePath(i))37:            **if** Cost(childNode) ≤ UpperBound **then**38:                childNode.vehicle ← $V_{i},\;$39:                childNode.vehicleTargetsList(i) ← [childNode.vehicleTargetsList(i)$T_{j}],$40:                childNode.targetsList ← parentNode.targetsList$\setminus T_{j}$41:                **if** childNode.targetsList =∅
**then**42:                    UpperBound ← Cost(childNode.cost)43:                    vehicleTargetsList(*i*) ← childNode.vehicleTargetsList(i)$i=1,\ldots ,N_{V}$44:                **else**45:                    OpenSet ← OpenSet ∪ childNode46:                **end**
**if**47:            **end**
**if**48:         **end**
**for**49:     **end**
**for**50:     OpenSet ← OpenSet ∖ parentNode51:**end**
**while**52:**return** vehicleTargetsList

The path length function returns the length of the relaxed path while avoiding obstacles between the vehicle’s current location and the target’s position. If the given time constraint is not satisfied, the path elongation algorithm is employed. If the path elongation algorithm is unable to satisfy the time constraint, the vehicle’s path length is given a value equal to infinity; this is done in order to ensure that the current branch is pruned, since the problem constraints are not fulfilled (Lines 12–14). The current target can now be removed from the targetsList of the expended branch (Line 16).

If a leaf node is reached (Line 17) and its cost is lower than the current upper bound (Line 18), the upper bound is updated to the cost of the current node (Line 19), and the vehicle’s assigned targets are stored in the vehicleTargetList variable (Line 20). Otherwise, the node is added to OpenSet for further exploration (Line 23).

After the initial nodes are generated, the exhaustive search begins. A depth first search is used to expand a branch until a leaf node is generated (Lines 27–52). While OpenSet is not empty, the last node inserted into OpenSet is chosen as the current parent node for further exploration, and the corresponding children nodes are created (Lines 29–49). Each child inherits the cost, vehicleTargetsList and vehiclePath fields from the parent node (Line 31). The child cost is calculated using the lostBenefitFunction, which is based on the vehicle’s accumulated path length (Lines 35–36). Additionally, the target’s time constraint is again compared to the vehicle’s accumulated path length, and if needed, the path elongation algorithm is used (Lines 32–34). If the child cost is lower than the current upper bound (Line 37), the child node’s vehicle, targetsList and vehicleTargetsList fields are updated accordingly (Lines 38–40). Otherwise, the branching is bounded.

As before, if the child node has an empty targetList (Line 41), then the UpperBound and the vehicleTargetsList are updated (Lines 42–43). If a leaf node is not reached and the child node targetList is not empty, the child node is added to OpenSet for further exploration (Line 45). Once evaluated, the parent node is removed from OpenSet (Line 50). This process is repeated until all branches have been either bounded or completely explored; OpenSet is empty. The algorithm output is a minimum cost ordered set of targets, assigned to each vehicle (Line 52). Owing to the tree search involved, this algorithm has an exponential time complexity.

### 4.2. Greedy Task Assignment Algorithm

The proposed algorithm is based on a greedy search method that enables finding an assignment solution quickly and is described in Algorithm 3. Since the algorithm is greedy by nature, the objective function used is the reward function presented in Equation (7).

**Algorithm 3** The heuristic greedy algorithm for task assignment.
**Input:** Vehicles’ initial configuration (*V*) and constant speed, targets’ position and their visit requirements (*T*), obstacle vertices; positions, targets’ time constraint (tc)
**Output:** Vehicle target list - the targets assigned to each vehicle and the required visitation order.1:TargetsList ← $\{T_{j},\;j=1,\ldots ,N_{T}$|$T_{j}$ has a visit requirement}2:TimeConstraint ← $\{tc_{j},\;j=1,\ldots ,N_{T}$}3:vehicleTargetsList(*i*) ← 0, $i=1,\ldots ,N_{V}$4:vehicleTotalBenefit(*i*) ← 0, $i=1,\ldots ,N_{V}$5:accumulatedPathLength(*i*) ← 0, $i=1,\ldots ,N_{V}$6:**while** TargetsList ≠∅
**do**7:     VehiclePath$\left(V_{i},T_{j}\right)$=PathLength$\left(V_{i},T_{j}\right)$,  $\left(i,j\right)\in \left\{1,\ldots ,N_{V}\right\}\times \left\{1,\ldots ,N_{T}\right\}$8:     **if** TimeConstraint < (VehiclePath$\left(V_{i},T_{j}\right)$+accumulatedPathLength$\left(V_{i}\right)$)/VehicleSpeed$\left(V_{i}\right)$
**then**9:         PathLength$\left(V_{i},T_{j}\right)$=PathElongation$\left(V_{i},T_{j}\right)$10:     **end**
**if**11:     VehicleBenefit$\left(V_{i},T_{j}\right)$ ← BenefitFunction(PathLength$\left(V_{i},T_{j}\right)$+accumulatedPathLength$\left(V_{i}\right)$),
                              $\left(i,j\right)\in \left\{1,\ldots ,N_{V}\right\}\times \left\{1,\ldots ,N_{T}\right\}$12:     $\left(i^{*},j^{*}\right)\leftarrow \arg\max_{\left(i,j\right)\in \left\{1,\ldots ,N_{V}\right\}\times \left\{1,\ldots ,N_{T}\right\}}$VehicleBenefit$\left(V_{i},T_{j}\right)$
                  subject to: $T_{j}\in$ targetsList &$T_{j}\notin$ vehicleTargetsList(*i*)13:      vehicleTargetsList($i^{*}$) ← [vehicleTargetsList($i^{*}$) $T_{j^{*}}$]14:     VehiclePosition($V_{i^{*}}$) ← VehiclePosition($T_{j^{*}}$)15:     accumulatedPathLength$\left(V_{i}^{*}\right)$ ← accumulatedPathLength$\left(V_{i}^{*}\right)$ + PathLength$\left(V_{i}^{*},T_{j}^{*}\right)$16:     vehicleTotalBenefit$\left(V_{i}^{*}\right)$ ← vehicleTotalBenefit$\left(V_{i}^{*}\right)$ + VehicleBenefit$\left(V_{i}^{*},T_{j}^{*}\right)$17:     TargetsList ← TargetsList$\setminus T_{j^{*}}$18:**end**
**while**19:**return** vehicleTargetsList

The key idea behind the proposed task assignment algorithm is the following: each vehicle is assigned an associated reward, equal to the benefit value acquired by the vehicle until now. A vehicle is assigned to a target only if the benefit value acquired by traveling from its current location to the target is maximum for all vehicle-target pairs. The assigned vehicle is first required to visit the target to which it is assigned. Then, assuming that the vehicle is at the assigned target point and that the target point is already visited, the process is repeated; that is, finding the next target-vehicle pair that has the highest benefit value of all other target-vehicle pairs, and so on.

The inputs to the algorithm are the initial configuration and constant speed of the vehicles, the locations of the target points, information about the obstacles and the time constraint of each target.

In each stage of the proposed algorithm, the motion planning algorithm is used as a subroutine to calculate the vehicle’s feasible trajectory to the relevant target point. Thus, the complexity of Algorithm 3 can be either polynomial or exponential, depending on the motion planning subroutine used (heuristic or exhaustive). If real-time applications are considered, the A*-like motion planning heuristic algorithm should be used as a subroutine in Algorithm 3, leading to polynomial complexity.

## 5. Simulation Results

In this section, sample runs are used to demonstrate the presented algorithms and to explain the different parameters’ (vehicle type, benefit’s descent rate, *etc*.) influence on the obtained solution. In the first scenario, the path elongation algorithm is demonstrated. Since only a single target is considered in this scenario, the benefit issue is ignored, and the objective is to obtain a feasible path that satisfies the given time constraint. In all of the remaining scenarios, the task assignment algorithms (exhaustive or greedy) use the motion planning subroutine (exhaustive or heuristic) based on the relaxed Dubins distances; hence, the coupling of the problem is kept. The vehicles’ turn radius is set to 60 m (in most cases, except where noted otherwise), and the targets’ initial benefit values can be set between 1000 and 10,000 (values are presented in the figures next to each target as a numeral between one and 10).

### 5.1. Path Elongation Algorithm Demonstration

In the following scenario, an aerial vehicle needs to be assigned to a single target with a time constraint. The vehicle must wait until the time constraint is satisfied before visiting the target. Since the path elongation algorithm is divided into four sequential steps, four figures that present the working of each step are given. In each figure, the scenario is slightly altered to initiate the algorithm’s different steps. The vehicle’s arrival times at the target point for the different scenarios are summarized in Table 1.

**Table 1:** Path elongation results summary.

Figure #	Step #	Time Constraint (s)	Vehicle Trajectory Time (s)	Number of Cycles	Solution Time (s)
9a	Step #1	2000	2357	4	$3.6\times 10^{-5}$
9b	Step #2	2000	2357	4	$9.6\times 10^{-5}$
9c	Step #3	2000	2011	3	0.45
9d	Step #4	2000	2072	0	4

**Figure 9:**
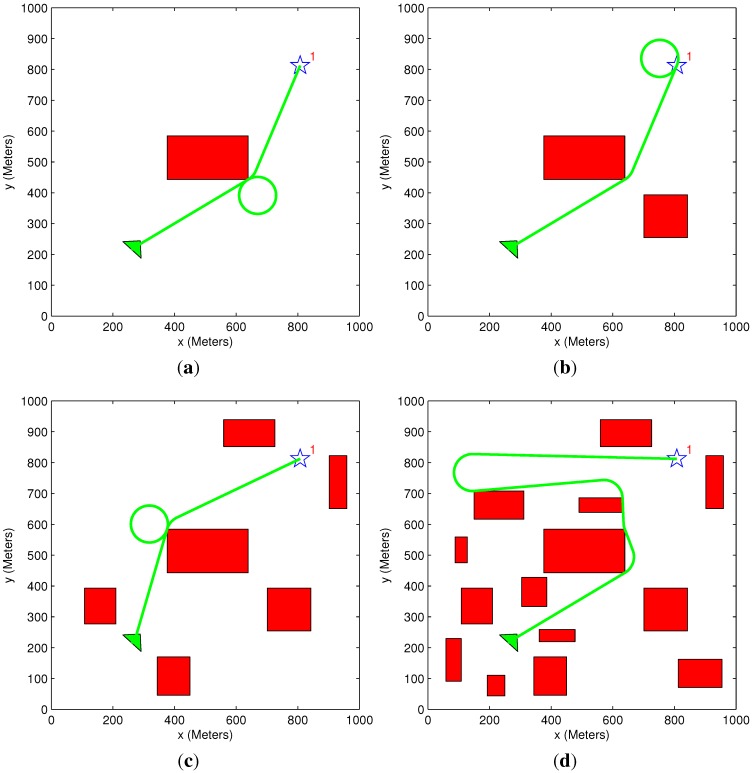
Path elongation demonstration. (**a**) Step #1; (**b**) Step #2; (**c**) Step #3; (**d**) Step #4.

In Figure 9a, the vehicle performs four loitering circles before heading towards the target. According to Step 1, the obstacle’s vertex, in which the circles are performed, is part of the elongation vertices list and is also one of the vehicle’s original path nodes (without the time constraint). In Figure 9b, an additional obstacle is added to the same scenario. Since in this case, the vertex in which the circles were previously performed is not part of the elongation vertices list, the vehicle performs four loitering circles around the target point. This is done according to Step 2. The scenario in Figure 9c includes additional obstacles, which initiate the third step of the elongation algorithm. Since the vehicle is unable to perform circles around any of the original path nodes, a new path is generated, which includes a direct relaxed path node and the target point. Three cycles are performed around the direct path node to satisfy the given time constraint. In Figure 9d, a new path is generated according to Step 4. In this specific scenario, loitering circles are not performed, even though the new path passes through an elongation vertex. This is due to the fact that the time constraint is already satisfied, as can be seen in Table 1.

### 5.2. General Scenario

**Figure 10:**
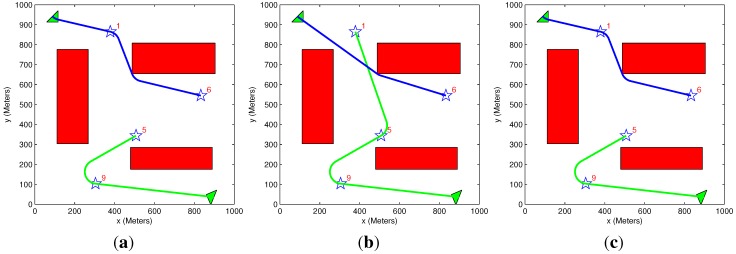
General scenario: two vehicles and four targets among obstacles. (**a**) Exhaustive task assignment algorithm; exhaustive motion planning algorithm; (**b**) greedy task assignment algorithm; heuristic motion planning algorithm; (**c**) exhaustive task assignment algorithm; heuristic motion planning algorithm.

Figure 10 presents a scenario in which two aerial vehicles need to visit four targets with different initial benefits. The scenario solution is obtained using different algorithm setups in each case. Table 2 summarizes the results of the different sample runs. The highest benefit (lowest lost benefit) and longest running time were gained using an exhaustive algorithm setup (Figure 10a). When heuristic motion planning is used instead (Figure 10c), the cost remains the same, but the running time decreases. In the case of a greedy task assignment and heuristic motion planning algorithms (Figure 10b), the lowest benefit (highest cost) and shortest running time are attained. The solution presented in Figure 10a demonstrates that the vehicles are generally first heading towards targets with high priority while taking into account targets with lower priority. Since the benefit diminishes with time, the vehicle does not head directly towards the high priority targets, but passes through low priority targets, which are closer to its location (upper vehicle on Figure 10a). In Figure 10b, the upper vehicles head directly to Target 6 (initial benefit value = 6) and skips Target 1, since, in this case, the task assignment algorithm is greedy by nature. In the scenarios presented in Section 5.3 and Section 5.4, the exhaustive algorithms’ setup yields the same results as the exhaustive task assignment algorithm and heuristic motion planning algorithm setup; therefore, only the latter setup is presented. Even though the results presented in these sections are identical, it can be shown that, in certain cases, the exhaustive algorithms’ setup provides better results, although the solution running time becomes longer.

**Table 2:** Different initial benefits scenarios.

Figure #	Algorithms Used	Initial Benefit	Acquired Benefit	Lost Benefit	Overall Distance	Solution Time (s)
10a	Exhaustive TAExhaustive MP	21,000	9918	11,082	1925	58.1
10b	Greedy TA Heuristic MP	21,000	9537	11,463	2423	1.05
10c	Exhaustive TA Heuristic MP	21,000	9918	11,082	1925	17.3

**Figure 11:**
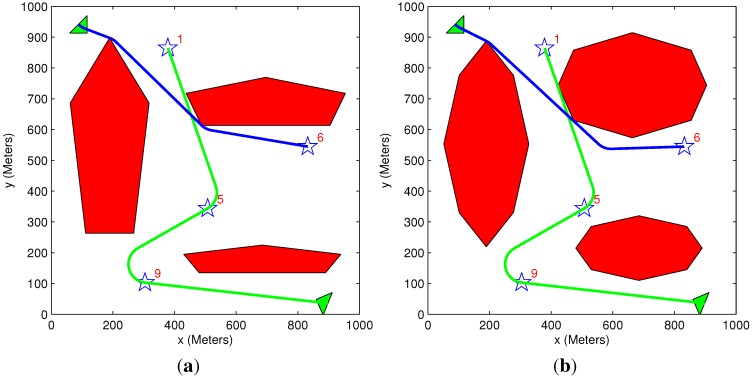
General scenario: two vehicles and four targets among different obstacle types; greedy task assignment algorithm and heuristic motion planning algorithm. (**a**) Pentagon obstacles; (**b**) Octagon obstacles.

Figure 11 presents a similar scenario to that of Figure 10, but with different polygon obstacles. Pentagon obstacles are present in Figure 11a, while octagon ones are in Figure 11b. Table 3 summarizes the results of the different sample runs. It can be seen that the type of obstacles has a negligible effect on the acquired benefit. In contrast, it significantly affects the run time of the algorithm, as having more edges enlarges the search space.

**Table 3:** Different initial benefits scenarios: pentagon and octagon obstacles.

Figure #	Algorithms Used	Initial Benefit	Acquired Benefit	Lost Benefit	Overall Distance	Solution Time (s)
11a	Greedy TA Heuristic MP	21,000	9479	11,521	2446	1.85
11b	Greedy TA Heuristic MP	21,000	9440	11,560	2462	2.73

### 5.3. Equal Benefit Scenario

**Figure 12:**
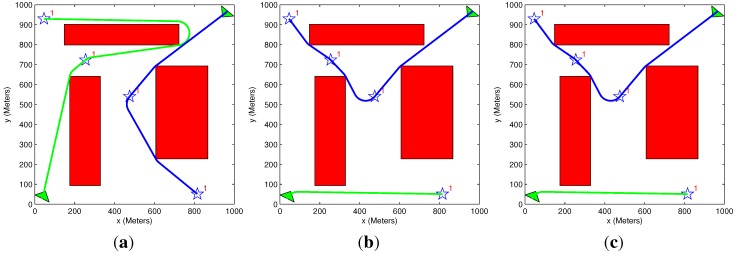
Equal benefits scenario: two vehicles and four targets among obstacles. (**a**) Greedy task assignment algorithm; heuristic motion planning algorithm; (**b**) exhaustive task assignment algorithm; heuristic motion planning algorithm; (**c**) exhaustive task assignment algorithm; exhaustive motion planning algorithm; minimize the sum of the overall distance traveled.

The scenario shown in Figure 12 is similar to the scenario shown in Figure 10, where only the targets’ initial benefit is equal. Since the targets’ priority is identical, it is expected that the results would be similar to the case where the cost function objective is to minimize the overall distance traveled by the vehicles (Equation (12)). In the results summarized in Table 4, the highest benefit (lowest cost) is obtained by the setup of the exhaustive task assignment algorithm (Figure 12b). The overall distance is the same as in the case of using the cost function, which minimizes the sum of the distance traveled (Figure 12c), as expected. As in the previous scenario, the solution running time has the same tendency, when using a greedy and heuristic algorithms’ combination to gain the shortest running time; with an exhaustive algorithms’ combination, the longest running time is gained. This tendency remains the same through all of the presented scenarios.

**Table 4:** Equal initial benefits scenario.

Figure #	Algorithms Used	Initial Benefit	Acquired Benefit	Lost Benefit	Overall Distance	Solution Time (s)
12a	Greedy TA Heuristic MP	4000	1399	2601	3353	1.3
12b	Exhaustive TA Heuristic MP	4000	1623	2377	2075	40.5
12c	Exhaustive TA Heuristic MP (Sum of path length cost function-Equation Equation (12))	4000	1623	2377	2075	40.5

### 5.4. Comparing Exhaustive and Greedy Task Assignment Algorithms

**Figure 13:**
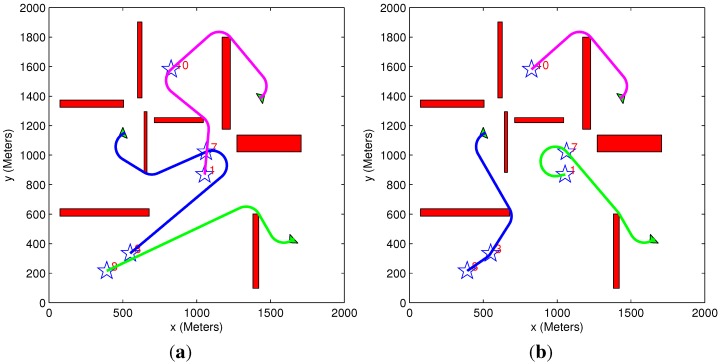
Comparison between exhaustive and greedy task assignment algorithms. (**a**) Greedy task assignment algorithm; heuristic motion planning algorithm; (**b**) exhaustive task assignment algorithm; heuristic motion planning algorithm.

A scenario involving three vehicles (each having a minimum turn radius of 100 m), five targets and eight obstacles is presented in Figure 13. The results of the scenario are given in Table 5. As expected, the exhaustive algorithm provides better or equal results compared to the greedy algorithm, at the expense of additional run time. This is also evident from Table 2 and Table 6.

**Figure 14:**
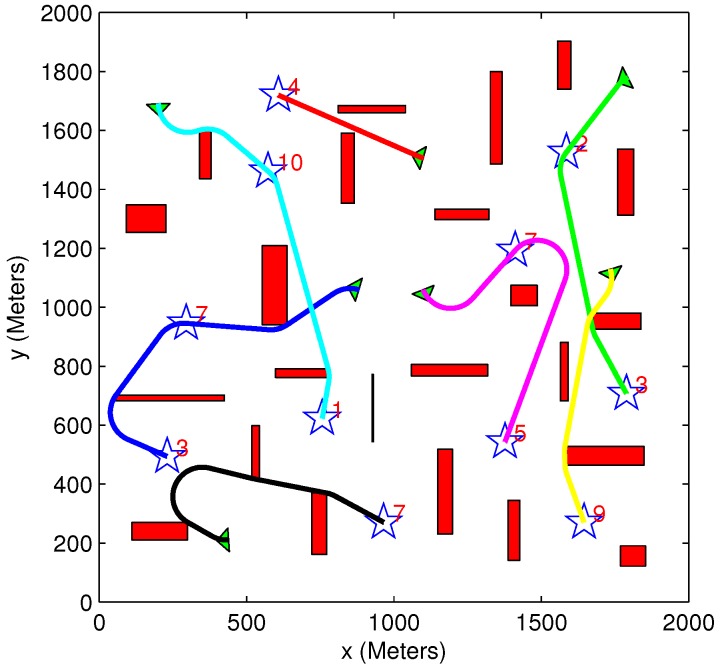
Complex scenario solved only using the greedy TA/MP algorithm.

The main advantage of the greedy algorithm is its low computational time, which makes it suitable for real-time applications. In cases where both the exhaustive task assignment and the motion planning algorithms are used, the best solution coded in the tree is obtained, and the lowest cost assignments allocation and vehicles’ paths are provided. However, due to the increased computational burden, such an algorithm may not be applicable for real-time application in a high dimensional problem and can mainly serve as a benchmark to evaluate (off-line) the performance of the greedy algorithm. Figure 14 presents the solution obtained using the greedy algorithm to a high dimensional problem involving seven vehicles, 11 targets and 23 obstacles. The run time of the algorithm was about three minutes, while the exhaustive algorithm did not return a solution within 24 h of run time. These computation times, as all other in this paper, were attained in a MATLAB implementation of the algorithm.

**Table 5:** Comparing exhaustive and greedy task assignment algorithms.

Figure #	Algorithms Used	Initial Benefit	Acquired Benefit	Lost Benefit	Overall Distance	Solution Time (s)
13a	Greedy TA Heuristic MP	29,000	20,120	8880	5340	5
13b	Exhaustive TA Heuristic MP	29,000	18,050	10,950	3520	150

**Table 6:** Scenario 1 and Scenario 2.

Figure #	Algorithms Used	Initial Benefit	Acquired Benefit	Lost Benefit	Overall Distance	Solution Time (s)
15	Exhaustive TA Exhaustive MP	15,000	6571	8529	1371	0.06
15	Greedy TA Heuristic MP	15,000	6571	8529	1371	0.016
16	Exhaustive TA Exhaustive MP	15,000	6634	8366	1333	0.06
16	Greedy TA Heuristic MP	15,000	6634	8366	1333	0.015

### 5.5. Benefit Time Dependency

**Figure 15:**
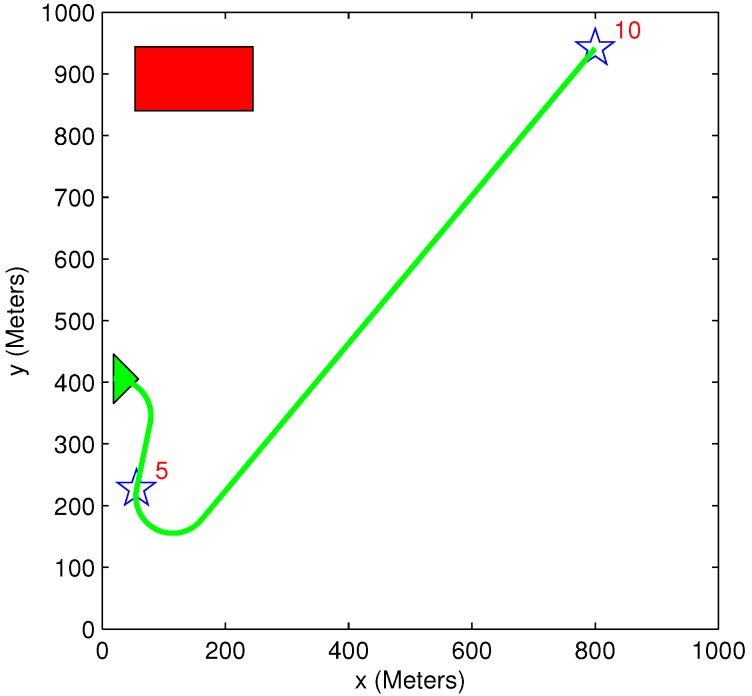
Scenario 1: Exhaustive TA; exhaustive MP and greedy TA; heuristic MP.

**Figure 16:**
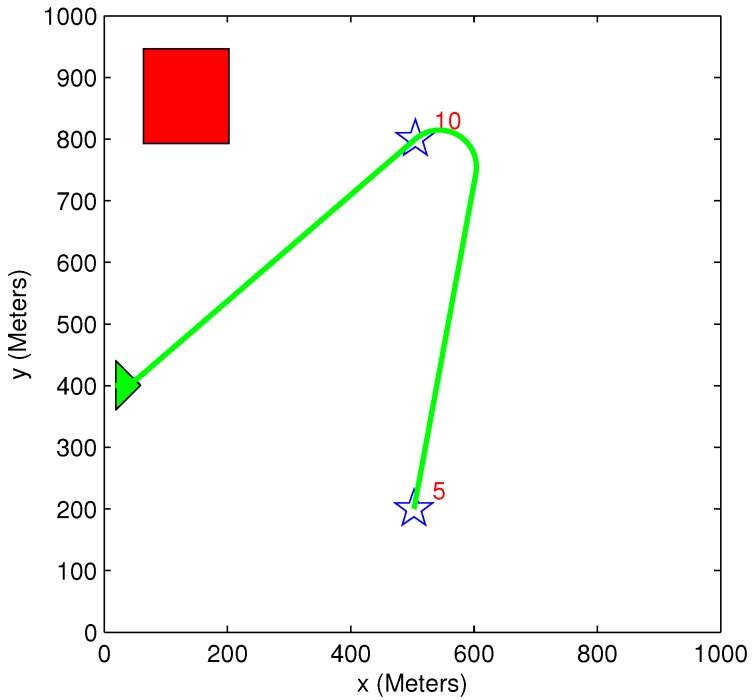
Scenario 2: Exhaustive TA; exhaustive MP and greedy TA; heuristic MP.

A simple scenario of one vehicle and two targets (initial benefit of 10 and 5) is used in Figure 15 and Figure 16. The time dependency can be easily explained using these two figures. In Figure 15, the vehicle’s path passes through Target 5, even though this causes the vehicle to extend its path toward Target 10. This happens because the time it takes to get to Target 5 is very short compared to Target 10, and it is better to first pass through Target 5 to minimize the “lost benefit” of the two targets. In Figure 16, however, the time it takes to get to Target 5 is still shorter than the time it takes to get to Target 10, but since Target 10 is now located closer to the vehicle, it is better to first pass through Target 10. These two scenarios demonstrate how the arrival time of the vehicle to each target influences the task assignment process. In both of these small-sized simple cases, the greedy algorithm provides an identical result to the exhaustive algorithm’s result, and the vehicle’s path remains the same.

### 5.6. Benefit Descent Rate

**Figure 17:**
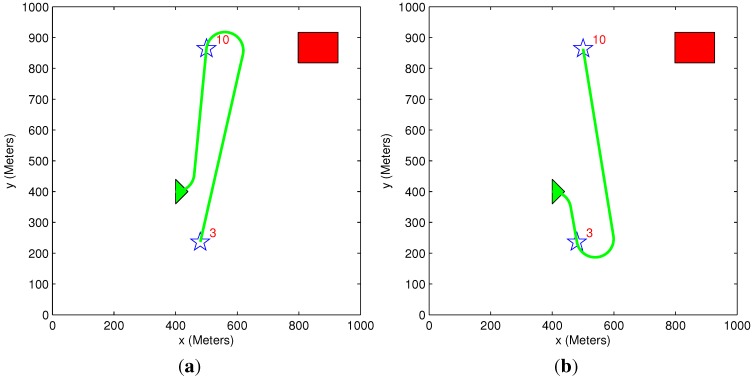
Benefit decent rate. (**a**) Exhaustive task assignment algorithm; exhaustive motion planning algorithm; decent rate: A = 0.001; (**b**) exhaustive task assignment algorithm; exhaustive motion planning algorithm; decent rate: A = 0.005.

The scenario of two targets and one vehicle presented in Figure 17 helps to illustrate the influence of the descent rate on the obtained results. In this scenario, the time it takes the vehicle to get to Target 3 is 200 s from its initial position, and the time it takes the vehicle to get to Target 10 is 500 s from the same position. By increasing the value of the descent rate, the benefit rapidly diminishes as time progresses. In Figure 17b, the descent rate is increased to A=0.005, five times compared to Figure 17a, causing a change in the vehicle assignments’ order (note that the threshold value for this change of assignment in the examined scenario was A=0.0034). Before the increase of the descent rate, the benefit of Target 10 is significantly higher than that of Target 3 (upper red bullet compared to lower red bullet in Figure 3a), but after the descent rate is increased, the targets have similar benefits (as can be seen by the red bullets’ vertical position in Figure 3b). Since the benefit that the vehicle can gather in Target 10 is smaller than the one in Target 3, it is preferable to change the targets’ visitation order, as can be seen in Figure 17b. The results summarized in Table 7 emphasize the influence of the descent rate, as the benefit acquired in Figure 17a is much higher than the one in Figure 17b. The benefit decent rate not only influences the benefit gathered, but may also influence the assignments’ order, as presented above.

**Table 7:** Benefit decent rate.

Figure #	Algorithms Used	Initial Benefit	Acquired Benefit	Lost Benefit	Overall Distance	Solution Time (s)
17a	Exhaustive TA Exhaustive MP	13,000	6866	6134	1321	0.05
17b	Exhaustive TA Exhaustive MP	13,000	1170	11,830	1004	0.05

## 6. Conclusions

In this paper, the intertwined problem of assigning and planning paths of UAVs to visit targets (having time-varying priorities) in an environment with obstacles was studied. It was assumed that the vehicles carry body-fixed sensors and, thus, are required to approach a designated target while flying straight and level. In order to address the time dependency of the targets’ priority, an objective function incorporating the feasible path length of the vehicles and the targets’ initial priority was formulated. Two task assignment algorithms were proposed: an exhaustive search algorithm that provides an optimal (lowest cost) solution and a greedy algorithm that provides a fast feasible solution (also used as an upper bound). A motion planning subroutine based on a tree search of an ordered set of targets and obstacles’ vertices is used as part of the task assignment solution and provides feasible vehicle paths. The targets’ time constraint was addressed by providing a path elongation algorithm amidst obstacles. Using simulations, the performance of the algorithms was compared, and the influence of the time-varying targets’ priority on the task allocation process was demonstrated and investigated. Although the greedy algorithm provides a sub-optimal solution, it is useful in large-scale real-time scenarios, where computational running time is of the essence. The exhaustive algorithm can provide an immediate solution that improves over run time for large-scale scenarios, or it can be used in off-line scenarios. It should be noted that as the Dubins model was used for representing the kinematics of UAVs, similar models may be used for representing the motion of other vehicles moving in a plane (such as ground vehicles), and thus, the developed motion and task assignment algorithms may be used.

## Appendix

The task assignment algorithm uses a motion planning subroutine, which is based on the study described in [12]. The motion planning subroutine includes two types of algorithms. These algorithms are based on a search process that explores the tree described in Section 3. The targets positions, vehicle’s initial configuration and obstacles vertices form the tree nodes set N. The two algorithms are described next, an exhaustive search algorithm that finds the minimum cost branch and a heuristic greedy algorithm that finds a feasible solution in a shorter computational time.

### ***A. Heuristic Motion Planning Algorithm***

The algorithm is given in Algorithm A1. Given a set of ordered targets, the vehicle’s initial configuration and the obstacle vertices’ position, the algorithm performs an A*-like greedy search based on Euclidean distance heuristics. A Euclidean distance between two points exists if and only if the straight line connecting the points does not intersect with the interior of an obstacle. The algorithm steps are as follows:

1. The vehicle is assumed to be located and oriented according to the initial configuration. A visit order list is initialized to null set (Line 1).
2. The following Steps 3–7 are repeated until the entire targets’ set is visited (Line 2).
3. The next point of the visit is chosen as $n^{*}$ until an obstacle-free relaxed path is found from the current point to the next target in the ordered targets’ set (Line 4).
4. $n^{*}$ is the node with the following property: the sum of the relaxed path length (connecting the vehicle’s current configuration and $n^{*}$) and the shortest Euclidean distance (connecting $n^{*}$ and the current target) is minimum (Line 5).
5. The node $n^{*}$ is added to the visit order list (Line 6).
6. The vehicle is assumed to be located at node $n^{*}$ (Line 7).
7. The current orientation angle becomes the arrival angle at $n^{*}$ of the relaxed path from the previous point to $n^{*}$ (Line 8).

The algorithm output is a vehicle trajectory, represented by an ordered set of nodes (including targets and obstacles’ vertices) that need to be visited using relaxed paths.

**Algorithm A1** Heuristic algorithm for motion planning of a Dubins vehicle amidst obstacles and multiple targets.	
	**Input:** Initial position and orientation of vehicle; obstacle information; ordered targets
	**Output:** Vehicle trajectory (visit order)
1:	Initialize: currentPosition ← startPosition; currentOrientation ← startOrientation; visitOrderList ← [ ]
2:	**for** iTarget = 1 to nTargets **do**
3:	$n^{*}$ ← currentPosition
4:	**while** $n^{*}\neq \text{iTarget}$ **do**
5:	$n^{*}$ ← $\arg\min_{n\in N}$ (RelaxedPath(currentPosition,currentOrientation,*n*) +
	ShortestEuclideanDistance(*n*,iTarget))
	subject to: relaxed path from currentPosition to *n* is obstacle free
6:	visitOrderList ← [visitOrderList $n^{*}$]
7:	currentPosition ← $v^{*}$
8:	currentOrientation ← final angle of relaxed path from currentPosition to $n^{*}$
9:	**end** **while**
10:	**end** **for**

### ***B. Exhaustive Motion Planning Algorithm***

The algorithm explores every branch of the search tree and evaluates every possible visit order sequence to find the minimum cost branch. The algorithm’s input is a set of ordered targets, the vehicle’s initial configuration and the obstacle vertices’ position. The algorithm steps are as follows:

1. Calculate the initial upper bound, using the heuristic algorithm (Line 1).
2. An OPEN list is generated to store the nodes that will be examined as the next node to visit.
3. The initial configuration is entered to OPEN as a node (Line 3).
4. The node with the lowest cost in OPEN (minimum relaxed path connecting the initial configuration and the node) is selected as the current node (Line 5).
5. The neighbors of the current node that can come after it in the visit order are examined (Line 7). Their estimated distance is defined as the sum of the following (Line 8):
  (a) Cost of the selected node.
  (b) Relaxed path length connecting the selected node and the neighbor.
  (c) Euclidean distance between the neighbor and the current target to visit.
  (d) The total Euclidean distance that connects the current target and the remaining targets to visit in the targets’ set in the required order.
6. The neighbors with an estimated distance that is lower than the current upper bound are added to OPEN as new nodes (Lines 9–10).
7. All of the new nodes added to OPEN are examined.
  (a) In the case a new node is the next target to visit, the target is marked as visited in the current explored branch (Line 12).
  (b) In the case a new node is the last target to visit and the entire targets’ set is visited in the required order, a leaf node of the branch is reached, and the entire branch is explored (Line 13).
    - The upper bound is updated to the relaxed path total length described by the nodes in the branch, and the visit order of the nodes is stored (Lines 14–15).
8. The current node is removed from OPEN, since all of the neighbors have been evaluated (Line 23).
9. This process is repeated until the OPEN list is empty.

**Algorithm B1** An exhaustive search algorithm for the motion planning of a Dubins/Reeds–Shepp vehicle amidst obstacles.	
	**Input:** Initial position and orientation of vehicle, obstacle information and ordered waypoints
	**Output:** Minimum length vehicle trajectory (visit order) and its length
1:	UPPER ← upper bound on the path length obtained from heuristic
2:	initialNode.{position ← initial position, angle ← initial orientation, vertex ← 0,
	targetsVisited ← 0, visitOrder ← [ ], cost ← 0}
3:	OPEN ← initialNode
4:	**while** notEmpty(OPEN) **do**
5:	currNode ← $\arg\min_{\text{O}PEN}$ OPEN.cost
6:	nextTarget ← currNode.targetsVisited+1
7:	**for** iNode = [nextTarget obstacleVertices] **do**
8:	pathLengthEstimate ← currNode.cost
	+ relaxedLength(currNode.{position,angle},iNode)
	+ EuclideanDistance(iNode,nextTarget)
	+ EuclideanDistanceCostToGo(nextTarget)
9:	**if** pathLengthEstimate < UPPER **then**
10:	newNode.{position ← position(iNode),
	angle ← arrival angle of relaxed path at iNode,
	vertex ← iNode, visitOrder ← [currNode.visitOrder iNode],
	cost ← currNode.cost
	+ relaxedLength(currNode.{position,angle},iNode)}
11:	**if** iNode = nextTarget **then**
12:	newNode.targetsVisited ← nextTarget
13:	**if** iNode = lastTarget **then**
14:	UPPER ← newNode.cost
15:	visitOrder ← newNode.visitOrder
16:	**end** **if**
17:	**else**
18:	newNode.targetsVisited ← currNode.targetsVisited
19:	**end** **if**
20:	OPEN ← OPEN ∪ newNode
21:	**end** **if**
22:	**end** **for**
23:	OPEN ← OPEN ∖ currNode
24:	**end** **while**

The algorithm output is identical to the heuristic algorithm output described above.

## References

[1] Cormen T.H., Leiserson C.E., Rivest R.L., Stein C. (2001). Introduction to Algorithms.

[2] LaValle M.S. (2006). Planning Algorithms.

[3] Shima T., Rasmussen J. (2009). UAV Cooperative Decision and Control: Challenges and Practical Approaches.

[4] Enright J., Savla K., Frazzoli E., Bullo F. (2009). Stochastic and Dynamic Routing Problems for Multiple Uninhabited Aerial Vehicles. AIAA J. Guid. Control Dyn..

[5] Shanmugavel M., Tsourdos A., Zbikowski R., White B. Path Planning of Multiple UAVs Using Dubins Sets. Proceedings of the AIAA Guidance, Navigation, and Control Conference.

[6] Dubins L.E. (1957). On curves of minimal length with a constraint on average curvature, and with prescribed initial and terminal positions and tangents. Am. J. Math..

[7] Chitsaz H., LaValle S.M. Time-optimal paths for a Dubins airplane. Proceedings of the 2007 46th IEEE Conference on Decision and Control.

[8] Agarwal P.K., Wang H. (2001). Approximation algorithms for curvature-constrained shortest paths. SIAM J. Comput..

[9] Backer J., Kirkpatrick D. A Complete approximation algorithm for shortest bounded-curvature paths. Proceedings of the 19th International Symposium on Algorithms and Computation.

[10] Jacobs P., Canny J. Planning smooth paths for mobile robots. Proceedings of the IEEE International Conference on Robotics and Automation.

[11] Laumond J.P., Jacobs P., Taix M., Murray R. (1994). A motion planner for nonholonomic mobile robots. IEEE Trans. Robot. Autom..

[12] Gottlieb Y., Manathara J.G., Shima T. (2015). Multi-Target Motion Planning Amidst Obstacles for Aerial and Ground Vehicles. Robot. Auton. Syst..

[13] Snape J., Manocha D. Navigating multiple simple-airplanes in 3D workspace. Proceedings of the 2010 IEEE International Conference on Robotics and Automation (ICRA).

[14] Yang K., Sukkarieh S. Real-time continuous curvature path planning of UAVS in cluttered environments. Proceedings of the 5th International Symposium on Mechatronics and its Applications.

[15] Yang K., Sukkarieh S. (2010). An Analytical Continuous-Curvature Path-Smoothing Algorithm. IEEE Trans. Robot..

[16] Shima T., Rasmussen S., Sparks A., Passino K. (2006). Multiple task assignments for cooperating uninhabited aerial vehicles using genetic algorithms. Comput. Oper. Res..

[17] Richards A., Bellingham J., Tillerson M., How J.P. Coordination and Control of Multiple UAVs. Proceedings of the AIAA Guidance, Navigation, and Control Conference, AIAA Paper 2002-4588.

[18] Schumacher C., Chandler P.R., Pachter M., Pachter L.S. (2007). Optimization of air vehicles operations using mixed-integer linear programming. J. Oper. Res. Soc..

[19] Chandler P.R., Pachter M., Rasmussen S.J., Schumacher C. Multiple task assignment for a UAV team. Proceedings of the AIAA Guidance, Navigation, and Control Conference.

[20] Schumacher C.J., Chandler P.R., Rasmussen S.J. Task allocation for wide area search munitions. Proceedings of the American Control Conference.

[21] Edison E., Shima T. (2011). Integrated task assignment and path optimization for cooperating uninhabited aerial vehicles using genetic algorithms. Comput. Oper. Res..

[22] Rasmussen S.J., Shima T. (2008). Tree search algorithm for assigning cooperating UAVs to multiple tasks. Int. J. Robust Nonlinear Control.

[23] Shima T., Rasmussen S., Gross D. (2007). Assigning micro UAVs to task tours in an urban terrain. IEEE Trans. Control Syst. Technol..

[24] Schumacher C., Chandler P., Pachter M., Pachter L. (2003). UAV Task Assignment with Timing Constraints.

[25] Schumacher C., Chandler P.R., Rasmussen S.J., Walker D. (2003). Path Elongation for UAV Task Assignment.

[26] Karaman S., Frazzoli E. (2011). Sampling-based algorithms for optimal motion planning. Int. J. Robot. Res..

[27] Kavraki L., Svestka P., Latombe J., Overmars M. (1996). Probabilistic roadmaps for path planning in high-dimensional configuration spaces. IEEE Trans. Robot. Autom..

[28] Donald B., Xavier P., Canny J., Reif J. (1993). Kinodynamic motion planning. J. ACM (JACM).

[29] Delle Fave F., Rogers A., Xu Z., Sukkarieh S., Jennings N. Deploying the max-sum algorithm for decentralised coordination and task allocation of unmanned aerial vehicles for live aerial imagery collection. Proceedings of the 2012 IEEE International Conference on Robotics and Automation (ICRA).

[30] Jiang L., Zhang R. (2011). An autonomous task allocation for multi-robot system. J. Comput. Inf. Syst..

[31] Shetty V., Sudit M., Nagi R. (2008). Priority-based assignment and routing of a fleet of unmanned combat aerial vehicles. Comput. Oper. Res..

[32] Krishnamoorthy K., Casbeer D., Chandler P., Pachter M., Darbha S. UAV search and capture of a moving ground target under delayed information. Proceedings of the 2012 IEEE 51st Annual Conference on Decision and Control (CDC).

[33] Shaferman V., Shima T. (2008). Unmanned aerial vehicles cooperative tracking of moving ground target in urban environments. AIAA J. Guid. Control Dyn..

[34] Boissonnat J.D., Bui X.N. (1994). Accessibility Region for a Car That Only Move Forward along Optimal Paths.

